# Murine Model for *Fusarium oxysporum* Invasive Fusariosis Reveals Organ-Specific Structures for Dissemination and Long-Term Persistence

**DOI:** 10.1371/journal.pone.0089920

**Published:** 2014-02-27

**Authors:** Katja Schäfer, Antonio Di Pietro, Neil A. R. Gow, Donna MacCallum

**Affiliations:** 1 Departamento de Genética, Universidad de Córdoba, Córdoba, Spain; 2 Aberdeen Fungal Group, Institute of Medical Sciences, Aberdeen, United Kingdom; University of Wisconsin Medical School, United States of America

## Abstract

The soil-borne plant pathogen *Fusarium oxysporum* causes life-threatening invasive fusariosis in immunocompromised individuals. The mechanism of infection in mammalian hosts is largely unknown. In the present study we show that the symptoms of disseminated fusariosis caused by *F. oxysporum* in immunosuppressed mice are remarkably similar to those reported in humans. Distinct fungal structures were observed inside the host, depending on the infected organ. Invasive hyphae developed in the heart and kidney, causing massive colonization of the organs. By contrast, chlamydospore-like survival structures were found in lung, spleen and liver. Systemically infected mice also developed skin and eye infections, as well as thrombosis and necrosis in the tail. We further show that *F. oxysporum* can disseminate and persist in the organs of immunocompetent animals, and that these latent infections can lead to lethal systemic fusariosis if the host is later subjected to immunosuppressive treatment.

## Introduction

Fungi of the genus *Fusarium* are important plant pathogens commonly found in soil, water and decaying organic matter [1]. In addition, *Fusaria* can cause a broad spectrum of diseases in humans, ranging from superficial or localized infections in healthy hosts to lethal disseminated fusarioses in immunocompromised patients [2]. Today, *Fusarium* is the second major cause of mould infections in immunocompromised patients after aspergillosis, and the incidence is increasing [3]. *Fusarium* species are among the most drug resistant fungal pathogens [4], [5], [6] and thus associated with high morbidity and mortality rates [7], [8]. Rapid diagnosis is essential for successful antifungal therapy and survival of the patient. In many cases the entry sites of disseminated *Fusarium* infections remain unclear, but reported portals of entry include onychomycosis, the respiratory tract (particularly paranasal sinuses), the gastrointestinal tract, and the central venous lines. However, few model systems for evaluating virulence and pathogenesis of this group of fungi have been described. Here we developed a murine infection model and use it to demonstrate that the infection symptoms caused by disseminated *Fusarium* infection in immunosuppressed mice are remarkably similar to those reported in humans [2], [3], [9]. One of the most frequent symptoms of infection by *Fusarium* species is the development of skin lesions, which are most commonly found on the extremities. They are described as painful subcutaneous nodules that can result in thrombosis and tissue necrosis [10]. Skin lesions are often the only source of diagnostic material, because blood cultures remain negative in many cases, in spite of blood dissemination [11]. Therefore, it is of interest to characterize the clinicopathological features of skin lesions and their role in *Fusarium* infection and to establish them in the diagnosis and management protocols of fusariosis. Previous studies suggest that *Fusarium* can cause skin lesions even in immunocompetent individuals, and that upon immunosuppression these foci can lead to the development of invasive and disseminated fusariosis [12]. It has been recommended that patients to undertake severe immunosuppressive therapy should undergo a thorough skin evaluation before initiation of therapy [9].

Soil-borne *Fusarium oxysporum* is the causal agent of vascular wilt, a devastating disease affecting a large variety of economically important crops worldwide [13]. *F. oxysporum* also causes invasive infections in immunosuppressed individuals, being the second most frequent species of the genus after *Fusarium solani,* [3], [14]. The incidence of invasive fungal infections leading to significant morbidity and mortality is rising because of the increase of the pool of immunocompromised patients caused by a dramatically increase number of solid organ transplants and the use of newer and more potent chemotherapeutic agents [15]. This is coupled with an increase in the number of reports of severe cases of invasive fusariosis often with lethal outcomes, due to the broad resistance to antifungal drugs [12], [16]. *F. oxysporum* infections are often misdiagnosed as a result of the histopathological similarities with *Aspergillus* or non-*Aspergillus* hyalohyphomycoses [2], [12], [17], [18].

Previous work established that a tomato pathogenic isolate of *F. oxysporum* f.sp. *lycopersici* can cause disseminated infection in immunocompromised mice [19]. The ability to cause disease in both plants and mammals makes *F. oxysporum* a unique multihost pathogen for studying fungal infection across different host kingdoms. To date several knockout mutants have been tested for virulence in immunosuppressed mice [19], [20], [21], [22], [23].

In the present study we used the *F. oxysporum*-mouse model to investigate infectious growth of *F. oxysporum* in mammals. We found that *F. oxysporum* develops distinct invasive structures, including hyphae, microconidia and chlamydospores, depending on the infected organ. We further show that *F. oxysporum* can cause thrombosis and necrosis in the tails of immunosuppressed mice, which can serve as model to study fungal skin infection in mammals. Finally we present strong evidence for dissemination of *F. oxysporum* in immunocompetent animals. Our results suggest that latent organ infections without macroscopic disease symptoms can initiate lethal systemic fusariosis, if the host is later subjected to immunosuppressive treatment.

## Methods

### *F. oxysporum* Isolate and Culture Conditions

*F. oxysporum* f.sp. *lycopersici* wild type strain 4287 was originally obtained from J. Tello, University of Almeria, Spain and stored as a glycerol microconidial suspension at −80 C. The pathogenicity of this wild type isolate on tomato plants is routinely confirmed by plant infection assays [24]. *Fusarium* conidia were cultured in potato dextrose broth at 28°C and 150 rpm for 4 days. For preparation of challenge inocula, microconidia were isolated by filtration as described previously [24], collected by centrifugation, washed, and resuspended in sterile physiological saline. The conidial concentration was adjusted with a hemocytometer to the desired density. The actual inoculum level was confirmed by plating serial dilutions on potato dextrose agar plates (PDA; Sigma) and incubating for 24 h at 28°C.

### Animal Infections

All animal experimentation was done in accordance with UK Home Office regulations and was approved by both the UK Home Office and the University of Aberdeen ethical review committee.

Female BALB/c mice (Harlan, UK; 6–8 weeks old) were maintained in groups of up to 6 animals per cage. All mice were fed sterilized laboratory chow and water *ad libitum*. Each animal was individually marked and was weighed daily. Immunosuppression was performed by intraperitoneal injection of 150 mg cyclophosphamide (Sigma) per kg body weight, which was repeated every 3 days thereafter. Depending on the experiment, the immunosuppressive treatment initiated either on day 3 prior to infection (day −3), on the same day of infection (day 0), or 3 or 7 days after infection (day 3 or 7, respectively).

Mice were infected by intravenous injection of 0.2 ml of a conidia suspension into the lateral tail vein. Mice were observed for up to 28 days post-challenge and were humanely terminated when they showed signs of severe illness and/or their body weight reduced by more than 20% of their initial body weight. Data were used to construct Kaplan-Meier survival curves, with differences determined by log rank statistics using GraphPad Prism 5.

### Tissue Burden and Histopathology

When mice were culled the hearts, lungs, kidneys, spleens and livers were aseptically removed, and one half of each organ was weighed and homogenized in 0.5 ml sterile saline. Ten fold serial dilutions of this homogenate were spread onto PDA. Plates were incubated at 28°C, colonies were counted after 48 h and CFU per gram for each organ were calculated. Fungal colony counts were converted to Log_10_ values and compared using the analysis of variance test. Data were analysed with the software GraphPad Prism 5.

The remaining halves of the organs were embedded in Cryo-M-bed (Bright Instruments, UK) and flash frozen. Sections (8 µm) were stained with Periodic acid-Schiff (PAS)-hematoxylin and examined by light microscopy. In addition, samples of spleen tissue from infected immunocompetent mice were homogenized, treated with KOH and stained with 25 µg ml^−1^ Calcofluor White (CFW) to visualize fungal structures.

## Results

### Effect of Inoculum Size and Timing of Immunosuppressive Treatment on the Severity of *F. oxysporum* Systemic Infection

**Figure 1:**
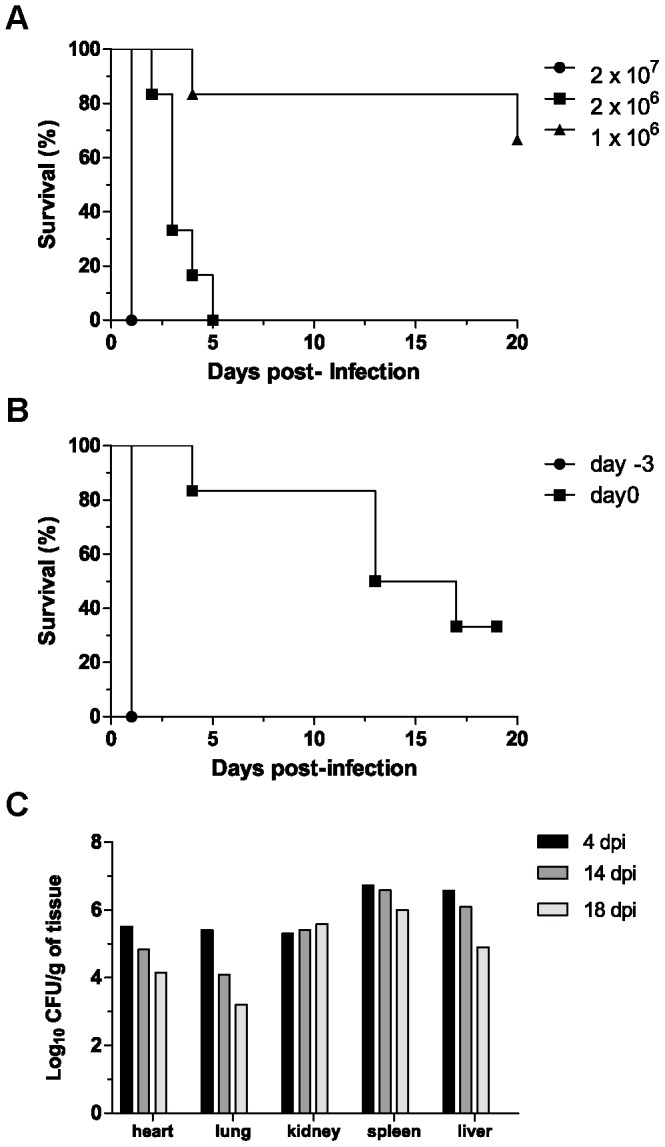
Effect of inoculum size and timing of immunosuppression on the severity of *F. oxysporum* infection. (A) BALB/c mice (n = 6) were inoculated intravenously with *F. oxysporum* microconidia. Immunosuppressive treatment (150 mg cyclophosphamide per kg body weight) was initiated three days prior to infection (day −3) and repeated every 3 days thereafter. Survival was recorded for 20 days. An inoculum dose of 2×10^6^ microconidia caused significantly lower mortality than 2×10^7^ microconidia (*p* = 0.0016); 10^6^ microconidia caused significant lower mortality than 2×10^6^ microconidia (*p* = 0.0024). (B) BALB/c mice (n = 6) were inoculated with 2×10^7^ microconidia, and immunosuppressive treatment was initiated either on day −3 or on day 0, and repeated every 3 days thereafter. Survival was recorded over 20 d. (C) Fungal burdens in mice immunosuppressed on day 0 and inoculated with 2×10^7^ microconidia. On each of the indicated days post-infection (dpi), one animal was sacrificed and organ homogenates quantified.

The effect of inoculum size on systemic infection by *F. oxysporum* in immunosuppressed mice was investigated. Intravenous inoculation with 2×10^7^ microconidia led to rapid development of systemic infection, with all mice becoming severely ill within 24 h (Fig. 1A). An inoculum of 2×10^6^ microconidia also caused 100% mortality, however disease progression was significantly slower (*p* = 0.0016), with all mice dying within 5 days. Finally, inoculation with 10^6^ microconidia caused a significant drop in mortality, with only two of the six infected mice succumbing to infection within the 20 d observation period (*p* = 0.0024).

We next tested the effect of timing of immunosuppression on disease severity. When the immunosuppressive treatment was initiated 3 d prior to infection (day −3) and repeated every 3 days thereafter, all mice succumbed to infection (Fig. 1A & B). By contrast, a delay in the start of the immunosuppression (day 0) led to a significant reduction in mortality (*p* = 0.0009) (Fig. 1B). Fungal burdens in the heart, lung and liver declined by 1–2 orders of magnitude over 18 days following inoculation, but remained at the same level in the kidneys and declined only slightly in the spleen (Fig. 1C).

### *F. oxysporum* Displays Distinct Invasion Strategies in Different Organs of the Host

**Figure 2:**
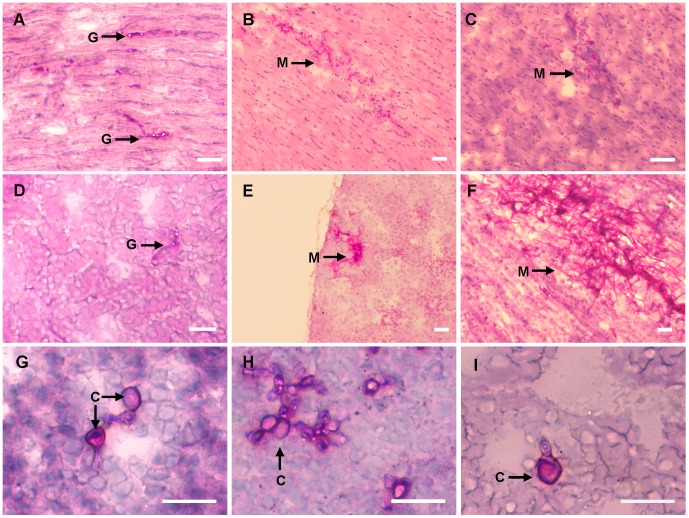
*F. oxysporum* displays distinct growth morphologies in different organs. PAS-hematoxylin-staining of tissue sections from different organs. Mice were subjected to immunosuppressive treatment on day 0, and every 3 days thereafter. Organs were obtained on day 1 (A, D), 4 (B, E, G, H, I) or 14 (C, F) after infection with 2×10^7^ microconidia: heart (A–C), kidney (D–F), lung (G), spleen (H), liver (I). Fungal structures are indicated by arrows. G, microconidial germlings; M, mycelium; C, chlamydospores. Scale bar = 50 µm.

**Figure 3:**
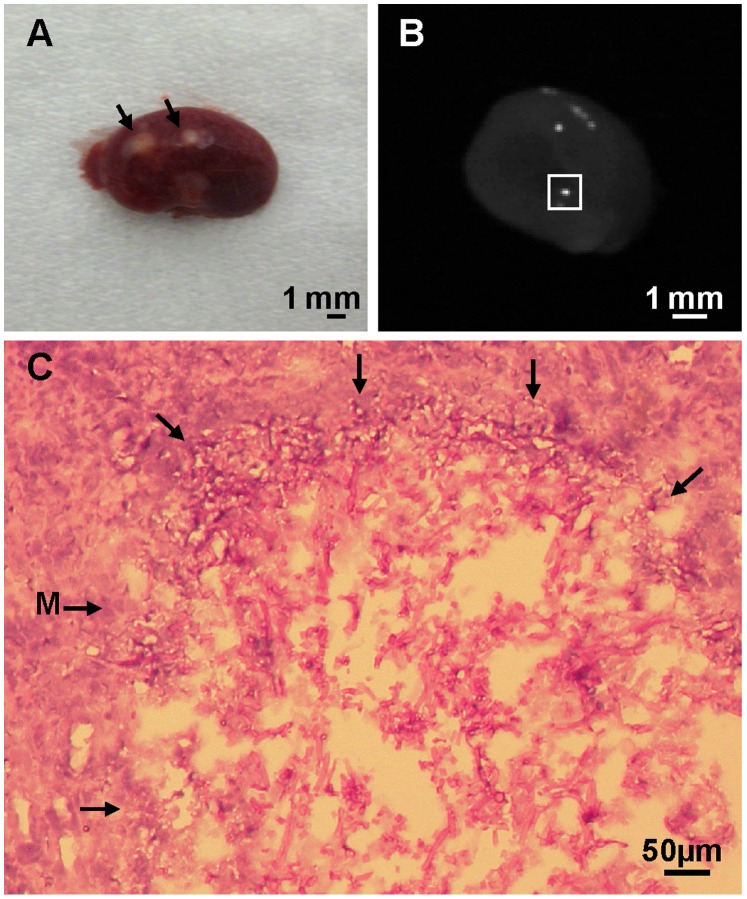
Invasive growth of *F. oxysporum* in the kidney. (A) Fungal biomass on the surface of an aseptically removed kidney from a mouse subjected to immunosuppressive treatment on day 0, and every 3 days thereafter. The kidney was obtained 20 d after infection with 10^6^ microconidia. Visible fungal lesions are indicated by arrows. (B) Kidney cross section from a mouse subjected to immunosuppressive treatment on day 0, and every 3 days thereafter. The kidney was obtained 20 d post-infection with 10^6^ microconidia. Bright areas correspond to fungal biomass stained with the chitin-binding dye Calcofluor White (CFW). The white box indicates the area used for the tissue section shown in C. (C) PAS-hematoxylin staining of a tissue section. The area of invasive mycelial growth is surrounded by arrows. M, mycelium. Scale bars are indicated.

Variations in the fungal burdens observed (Fig. 1C) prompted us to conduct a histopathological analysis of the invasive growth of *F. oxysporum* in mice subjected to immunosuppressive treatment on day 0, and every 3 days thereafter. PAS-hematoxylin-staining of tissue sections revealed striking differences in fungal development between different organs. At 24 h post inoculation, a significant number of microconidia had germinated in the heart and kidney (Fig. 2A & D), whereas only ungerminated microconidia were observed in lung, spleen and liver (data not shown). After 4 d post-inoculation (dpi), branched fungal hyphae had developed in the heart and kidney (Fig. 2B & E), leading to the production of large mycelial colonies and the complete invasion of the infected tissue area at 16 dpi (Fig. 2C & F). Invasive growth was particularly aggressive in the kidneys (Fig. 3), where the fungal biomass was detectable macroscopically on the surface of the organ (Fig. 3A & C). By contrast, invasive growth was not detected in the lung, spleen and liver. Instead, *F. oxysporum* developed chlamydospore-like structures, which were detected at 4 dpi and remained visible at 16 dpi (Fig. 2G, H & I).

### *F. oxysporum* Causes Thrombosis and Necrosis in Tails and Infections in Eyes of Immunosuppressed Mice

**Figure 4:**
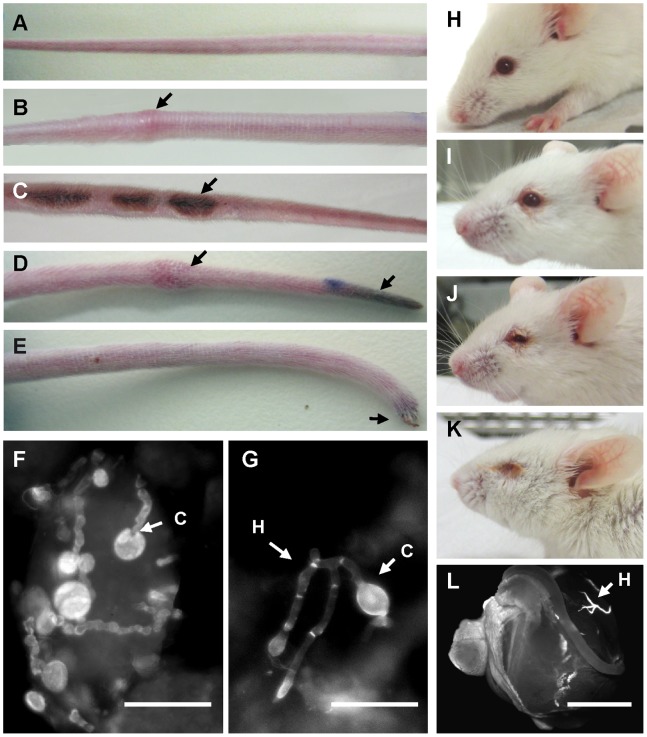
*F. oxysporum* causes infection in the tails and eyes of immunosuppressed mice. Mice were subjected to immunosuppressive treatment on day 0, and every 3 days thereafter. (A) Tail of a non-infected immunosuppressed BALB/c mouse. (B–E) Tails of immunosuppressed BALB/c mice intravenously inoculated with 10^6^ conidia at 10 d post-infection. Macroscopic symptoms are indicated by arrows: swellings (B, D), open lesions (C), necrosis (D, E), and loss of the tip (E). (F, G) CFW staining of tissue samples taken from the lesion shown in (C). Fungal structures are indicated by arrows. H, hyphae; C, chlamydospores. (H–L) Infection of the eye tissue in immunosuppressed mice infected with *F. oxysporum*. (H) Uninoculated mouse; (I–K) mice inoculated with 10^6^ conidia at 10 dpi. (L) CFW staining of an eye removed from an immunosuppressed mouse infected with *F. oxysporum.* H, hyphae. Scale bar = 50 µm (F, G); 1 mm (L).

We noted that the immunosuppressed mice infected with *F. oxysporum* developed bulges and swellings which appeared at multiple sites along the tail, not restricted to the site of inoculation (Fig. 4B). Some of these swellings subsequently turned into open lesions (Fig. 4C & E). In addition, some mice displayed macroscopic symptoms of necrosis at the tail. The necrotic tissue was initially visible as a black region at the tip (Fig. 4D & E) which spread upwards along the tail, in some cases leading to loss of the tip (Fig. 4F). Calcofluor white (CFW) staining of tissue samples obtained from the open wounds revealed the presence of chlamydospores and hyphae of *F. oxysporum* (Fig. 4G & H).

Another observation associated with the presence of *F. oxysporum* in the immunosuppressed mice was the development of infection symptoms around and within the eye (Fig. 4I–M). The presence of fungal structures in the infected eye tissue was confirmed microscopically (Fig. 4N).

### *F. oxysporum* Disseminates and Persists in Immunocompetent Mice

**Figure 5:**
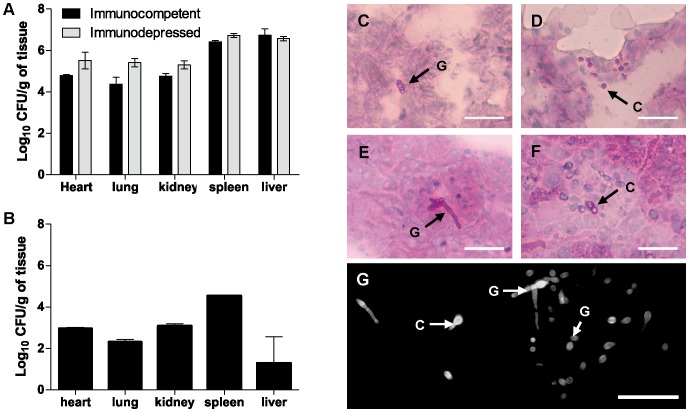
*F. oxysporum* disseminates and persists in immunocompetent mice. (A) Fungal burdens were determined at day 4 post-infection for groups of 3 BALB/c mice, either immunocompetent or immunosuppressed on day 0 and every 3 days thereafter, and intravenously infected with 2×10^7^ microconidia. (B) Fungal burdens in infected immunocompetent mice sacrificed at 11 d post-infection. (C–F) PAS-hematoxylin-staining of tissue sections obtained from different organs of immunocompetent mice sacrificed at 4 d post-infection. (C) Heart, (D) lung, (E) kidney, (F) liver. (G) Spleen sample from an infected immunocompetent mouse sacrificed at 11 d post-infection was homogenized, KOH-treated and stained with CFW to visualize fungal structures (indicated by arrows). G, microconidial germlings; C, chlamydospores. Scale bar = 50 µm.

Inoculation *F. oxysporum* typically leads to 70–100% mortality in immunosuppressed mice, whereas immunocompetent animals fail to display detectable signs of illness ([19] and this work, data not shown). Thus, we investigated whether *F. oxysporum* was able to disseminate and persist in an immunocompetent host. Strikingly, fungal burdens at 4 dpi in immunocompetent mice inoculated with 2×10^7^ microconidia were similar or only slightly lower than in animals subjected to immunosuppressive treatment on day 0, and every 3 days thereafter (Fig. 5A). *F. oxysporum* microconidia and germlings were observed in the heart, lung, kidney, spleen and liver of immunocompetent animals (Fig. 5C–F). At 11 dpi the fungal burdens in immunocompetent animals had declined by approximately two orders of magnitude in most of the organs, but remained relatively high (∼10^5^ CFU per g tissue) in the spleen (Fig. 5B). Indeed, PAS-hematoxylin and CFW staining confirmed the presence of chlamydospores, as well as germinated and ungerminated microconidia in different organs of infected animals at 4 and 11 dpi (Fig. 4G).

### Persistence of *F. oxysporum* in the Immunocompetent Host can Lead to Subsequent Systemic Infection upon Immunosuppressive Treatment

**Figure 6:**
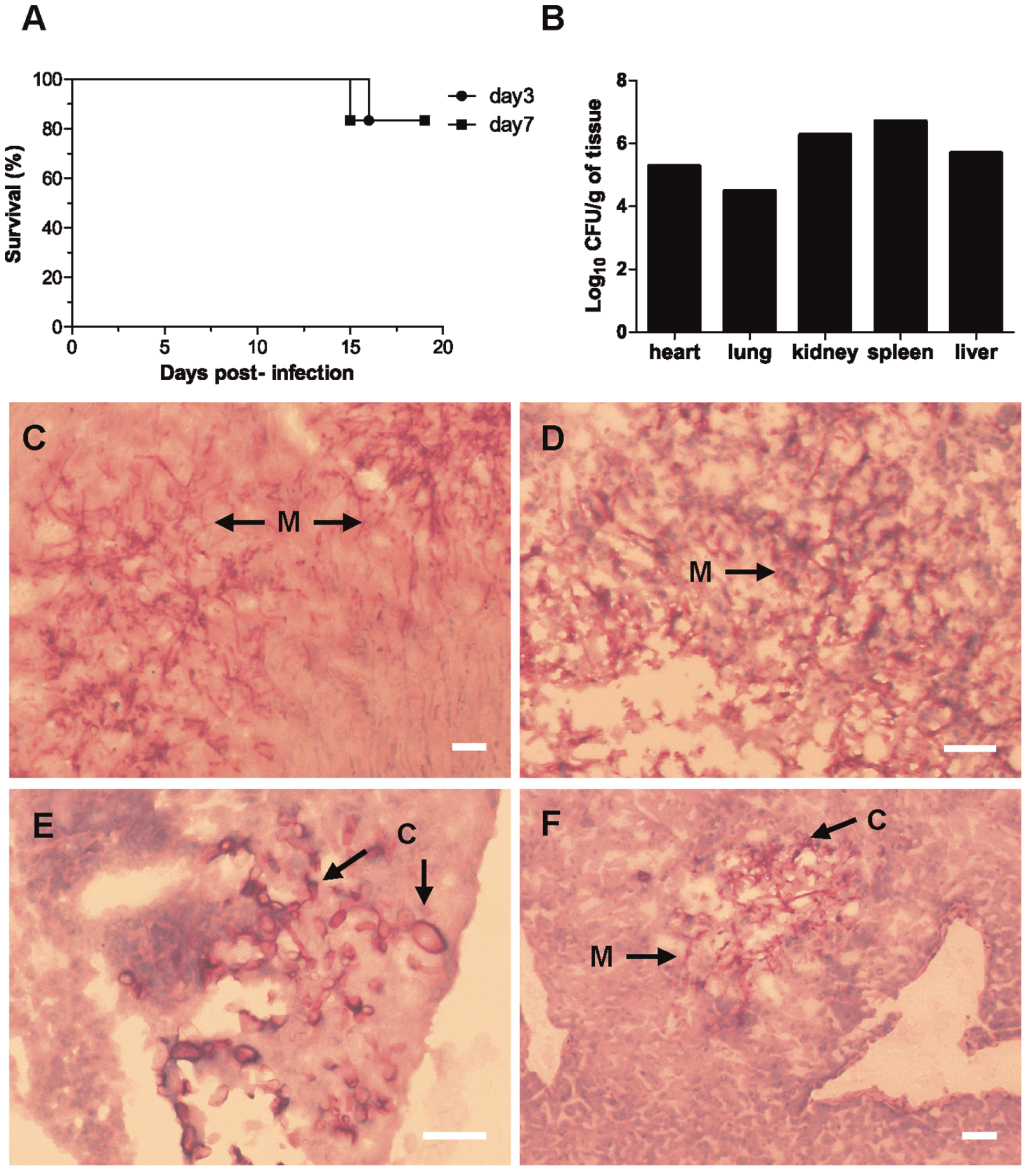
*F. oxysporum* persistence in immunocompetent mice can lead to systemic infection and death upon subsequent immunosuppression. (A) BALB/c mice (n = 6) were intravenously inoculated with 2×10^7^ microconidia. Immunosuppressive treatment was initiated either on day 3 or 7 post-infection and repeated every 3 days thereafter. Survival was recorded over 20 d. Fungal burdens (B) and PAS-hematoxylin-staining (C–F) of organ sections from a mouse inoculated with 2×10^7^ *F. oxysporum* microconidia and subjected to immunosuppressive treatment starting at 7 dpi. The mouse that died was culled at 15 dpi. Organs: heart (C), kidney (D), spleen (E), liver (F). M, mycelium; C, chlamydospores. Scale bar = 50 µm.

The presence of fungal propagules in the organs of immunocompetent mice even at >10 dpi suggests that *F. oxysporum* can develop resting structures such as chlamydospores, but also actively growing mycelium to persist within the immunocompetent host. Such a host might subsequently become susceptible to invasive fusariosis if the immune system is disrupted. When immunocompetent mice inoculated with *F. oxysporum* were subjected to immunosuppressive treatments starting at either 3 or 7 dpi and repeated every 3 days thereafter, one mouse in each group died at 16 and 15 dpi, respectively (Fig. 6A). Fungal burden was determined in different organs from one of the killed mice, revealing high counts of fungal propagules (Fig. 5B). Tissue sections confirmed the presence of invasive mycelial growth in multiple organs, including heart, kidney spleen and liver, indicative of systemic fusariosis (Fig. 5C–F).

## Discussion

Fungi of the genus *Fusarium* are common soil inhabitants and notorious plant pathogens, and have long been recognized as etiologic agents of focal infections of the skin, nails, and cornea of humans [25]. Moreover, invasive *Fusarium* infections in immunosuppressed patients are associated with high mortality rates [7], [8]. Rapid antifungal therapy, achieved by a prompt diagnosis, is essential for survival of these patients. A critical step is the identification of *Fusarium,* which is often made difficult by some histopathological similarities with *Aspergillus.* Infections caused by both *Fusarium* and *Aspergillus* are characterized by hyaline branching septate hyphae at acute angles, which invade the blood vessels causing thrombosis and tissue infarction [18], [26]. Therefore, a detailed characterization of the invasive behaviour of *Fusarium* within the host, including growth and development of fungal structures formed by the pathogen and associated with disease, is required and will have important consequences for the diagnosis and management of fusariosis. Here we used the mouse model to investigate *Fusarium* infection in mammals. The usefulness of the animal model is highlighted by the finding that the disease symptoms observed in mice are remarkably similar to those reported in human fusariosis [2], [3], [9].

A key result of this study is that *F. oxysporum* displays distinct invasion strategies in different organs of mice. While the fungus initiated hyphal growth 24 h after inoculation in the kidney and the heart, no such growth was detected in the spleen, liver or lung. Instead, chlamydospore-like structures were formed in these organs. This novel finding represents an important advance in our understanding of *Fusarium* infections in mammals. Chlamydospores are thick-walled cells generally developed through the modification of hyphal and conidial cells. Their formation is induced by aging or unfavorable environmental conditions such as low temperatures or carbon starvation. Chlamydospores represent the principal structure for long-time survival during unfavorable periods in the soil, and play an important role as primary inoculum for plant root infection [27], [28], [29], [30], [31]. Our findings highlight the importance of detailed histopathological analysis of infection structures, in addition to routine determination of fungal burden. For example, kidneys contained reduced fungal burdens compared to the spleen, but microscopic analysis revealed massive mycelial invasive growth in this organ, which in some instances was even visible macroscopically on the organ surface. Thus, although fungal burden is a useful parameter for assessing fungal dissemination in the host, it fails to provide detailed information on the impact of filamentous pathogens on the infected organs. It was reported previously that during *F. solani* or *A. fumigatus* infection, quantitative culture led to underestimation of absolute fungal burden as compared to non-culture-based methods such as quantitative PCR or determination of galactomannan levels by enzyme immunoassays (EIAs) [32], [33], [34], [35]. In an inhalational rat model of invasive pulmonary aspergillosis (IPA), both real-time nucleic acid sequence-based amplification (NASBA) and qPCR showed a progressive increase of fungal biomass in lung tissue, whereas CFU counts were stable over time [36].

A frequent symptom associated with *Fusarium* infections is the development of superficial skin lesions, described as subcutaneous nodules that can result in thrombosis and tissue necrosis [10]. In more than 60% of disseminated *Fusarium* infections skin biopsies of affected patients revealed necrosis of skin papules [37] and microvessel thrombosis associated with fungal hyphae [10]. Here we found that infection of immunosuppressed mice by *F. oxysporum* led to macroscopic symptoms in the tail, including necrosis of the skin, swellings and wounds and even loss of the tail tip. Microscopic analysis of the skin lesions confirmed that the nodules contained both chlamydospores and hyphae of *F. oxysporum*. CFW staining proved to be highly useful for detection of progressive systemic fusariosis in the mouse system, and could therefore be used as a rapid diagnostic tool in immunocompromised patients. Because skin lesions often appear during early stages of *Fusarium* infection before the disease spreads to the trunk and the extremities, they are crucial for the diagnosis and management of fusariosis, allowing rapidly initiation of a specific treatment in order to prevent progression of skin lesions and further necrosis.

*F. oxysporum* keratitis is one of the most important causes of corneal ulcers, ocular morbidity and visual loss in developing nations [38]. It is also a common type of infection caused by *F. solani* in immunocompetent individuals worldwide [39], [40], [41], [42], [43], [44]. A previous study on disseminated *F. solani* infection via inoculation of microconidia in the lateral tail vein of immunocompetent mice reported disease symptoms both in intra-ocular structures and in neighbouring muscles [45] Here we provide evidence for the presence of fungal biomass around and within the eyes of immunosuppressed mice, associated with disseminated *F. oxysporum* infection. This result supports the view that fungal keratitis should be established as part of the diagnostic protocol for disseminated *Fusarium* infections in order to prevent a delay in diagnosis or inadequate treatment, which may lead to loss of the affected eye. Further, we observed that mice affected by disseminated *F. oxysporum* infection showed a “twister” phenotype indicating a possible infection of the brain. Supporting this idea, analysis of fungal burden in the brain of an animal displaying a twister phenotype showed the presence of fungal biomass (data not shown), suggesting that *F. oxysporum* is able to enter the mouse brain.

Initiation of immunosuppressive treatment at day −3 led to death of all infected animals, while mortality was significantly lower when immunosuppression was started in parallel to infection. Thus, the immune status of the host has a major effect on the severity of infection by *F. oxysporum*. Importantly, we show here for the first time that *F. oxysporum* can also disseminate and persist in immunocompetent individuals. Unexpectedly, fungal burdens in immunocompetent mice at 4 dpi were only slightly lower than in immunosuppressed animals, with microconidia and germlings observed in the heart, lung, kidney, spleen and liver of immunocompetent animals even after more than 10 dpi. The finding that *F. oxysporum* can persist in an immunocompetent mammalian host is highly relevant, because these fungal foci could lead to subsequent systemic infection upon immunosuppressive treatment. Indeed, one mouse in each group later succumbed to fungal infection. We conclude that invasive fusariosis in these animals was caused by chlamydospore-like structures which had persisted in the organs, suggesting that latent fungal survival structures have the potential to initiate invasive mycelial growth once the immune system is no longer effective.
